# Paper-Mulberry-Silage-Enhanced Lipid Metabolism and Milk Polyunsaturated Fatty Acid Concentration in Dairy Cows: A Multi-Omics Study

**DOI:** 10.3390/ijms27219940

**Published:** 2026-11-06

**Authors:** Shuaikang Yang, Weixuan Tang, Yajing Wang, Hongjian Yang, Wei Wang, Zhijun Cao, Wei Wang, Shengli Li, Yueyu Bai, Yangyi Hao

**Affiliations:** 1 Institute of Livestock Animal Breeding and Reproduction, School of Life Sciences, Henan University, Zhengzhou 450046, China; 2 State Key Laboratory of Animal Nutrition and Feeding, Beijing Engineering Technology Research Center of Raw Milk Quality and Safety Control, College of Animal Science and Technology, China Agricultural University, Beijing 100193, China; 3 School of Applied Science, Taiyuan University of Science and Technology, Taiyuan 030024, China; wangwei11@tyust.edu.cn

**Keywords:** paper mulberry, dairy cow, milk, polyunsaturated fatty acid, lipid metabolism

## Abstract

Developing alternative forages that modify milk fatty acid composition is important for improving milk nutritional quality and diversifying feed resources in dairy production. Previous studies have demonstrated the potential of paper mulberry (PM, *Broussonetia papyrifera*) silage to partially replace conventional roughages and influence antioxidant status and milk fatty acid composition. However, its effects on milk polyunsaturated fatty acids (PUFAs) and the associated ruminal microbial and metabolic mechanisms remain insufficiently characterized. This study aimed to evaluate the effects of feeding PM silage on the concentration of polyunsaturated fatty acids in milk, the structure of the rumen microbial community, and lipid metabolic functions, as well as to analyze the associations between rumen microbial and metabolic profiles and the fatty acid composition of milk. Thirty lactating Holstein cows were randomly assigned to control and PM groups, with 15 cows per group. The PM group received diets containing 9% PM silage for the first 28 days and 18% for the subsequent 28 days on a dry matter basis. Rumen fluid, plasma, and milk were collected on day 56 for integrated analyses combining fatty acid profiling, ruminal fermentation measurements, 16S rRNA gene sequencing, metagenomics, and metabolomics. Milk PUFA concentration was significantly higher in the PM group than in controls, with no significant differences in dry matter intake or milk yield. PM feeding also altered ruminal microbial community composition and lipid-metabolism-related functional gene abundances, accompanied by increased glycerophosphocholine and glycerophosphoethanolamine levels. Integrated analyses linked these changes to fatty acid profiles in rumen fluid, plasma, and milk, supporting ruminal lipid remodeling as a candidate mechanism underlying the increase in milk PUFA concentrations. These findings provide evidence for the microbial and metabolic basis of the effects of PM silage on milk fatty acid composition and inform its development as a functional forage and the optimization of dairy cow feeding strategies.

## 1. Introduction

Milk is a significant source of essential nutrients for humans, including calcium, phosphorus, vitamin D, and a wide variety of fatty acids (FAs) [1]. The functional and bioactive compounds in milk have attracted increasing attention due to their benefits for human health [2]. Among these, unsaturated fatty acids (UFAs) are of particular importance, accounting for approximately 30% of milk fat [3,4]. Milk contains ω-3 and ω-6 polyunsaturated fatty acids such as linoleic acid (LA), α-linolenic acid (ALA), γ-linolenic acid (GLA), eicosapentaenoic acid (EPA), and docosahexaenoic acid (DHA). These UFAs have been reported to reduce blood lipid levels, enhance antioxidant capacity and immunity, and help alleviate cardiovascular diseases. Therefore, dietary strategies for modifying milk FA composition, particularly PUFA concentrations, are of interest in dairy nutrition and milk quality research.

Milk PUFA enrichment through dietary modification is constrained by microbial lipid transformations in the rumen, which influence the availability of ingested UFAs for absorption and incorporation into milk [5,6]. The course of biohydrogenation is sensitive to dietary context, with changes in forage-to-concentrate ratio and lipid supplementation altering the formation of FA intermediates [7]. Thus, forage-related changes in milk PUFA may involve modifications of the ruminal environment as well as dietary lipid supply. Plant secondary metabolites provide an additional influence: condensed tannins can modify biohydrogenation in vitro [8], while flavonoid supplementation has altered ruminal fermentation and microbial communities in dairy cows [9]. However, milk FA responses to phytochemical supplementation vary across studies and feeding conditions [10,11,12,13]. Forage substitution introduces further complexity because nutrient supply and secondary metabolites may change simultaneously. Replacing grass silage with sainfoin silage reduced apparent ruminal biohydrogenation and increased feed-to-milk PUFA transfer [5], whereas different tanniferous forage legumes and cultivars elicited distinct changes in milk α-linolenic acid [14]. Ensiled paulownia leaves also increased milk PUFA proportions alongside changes in ruminal fermentation and microbial populations [15]. Collectively, these findings suggest that forage substitution can influence milk UFA composition and ruminal microbial community structure.

Paper mulberry (PM, *Broussonetia papyrifera*), a fast-growing tree of the Moraceae family native to East Asia, is considered a high-quality forage due to its high protein content [16,17,18]. PM provides dietary protein and fibrous plant material, and its feeding value varies with the plant fraction used. In particular, PM leaves have higher crude protein concentrations and greater digestibility than stems. PM has also been investigated as a forage resource containing bioactive constituents, including phenolic and flavonoid compounds [19]. Several studies have reported the potential benefits of PM in improving lactation performance and immunity in dairy cows. Wu et al. reported enhanced antioxidant capacity in dairy cows supplemented with PM silage [18]. Si et al. observed higher immunoglobulin and antioxidase levels in mid-lactation cows fed a diet containing 15% PM silage. Previous research has also reported increased milk PUFA concentrations in cows receiving diets containing 10–15% PM silage [20]. These findings provided a rationale for examining the ruminal microbial and lipid metabolic responses associated with PM feeding.

Despite these findings, the relationships between PM feeding, ruminal microbial lipid metabolic potential, and milk PUFA concentrations remain insufficiently characterized. Therefore, this study aimed to evaluate the effects of a PM silage feeding regimen on milk PUFA concentrations, ruminal microbial communities, and lipid metabolic potential, and to examine associations between ruminal microbial and metabolic features and milk FA composition. To address this objective, we characterized FA profiles in rumen fluid, plasma, and milk, as well as ruminal VFA profiles. We further combined 16S rRNA gene sequencing, metagenomics, and rumen metabolomics to characterize microbial community structure, functional potential, and metabolic profiles.

## 2. Results

### 2.1. Dietary Fatty Acid Intake and Milk Fatty Acid Yield

In the present study, diet supplemented with PM silage did not affect the feed intake, milk yield, or milk composition (Table S2). The fatty acid profile in the cows’ diet is shown in Table S3. The contents of UFA, PUFA, MUFA, and omega-6 fatty acid were lower in a PM-silage-based diet than in a normal diet. Dairy cows supplemented with PM silage had lower UFA, PUFA, MUFA, and omega-6 fatty acid daily intake than dairy cows in the CON group (Table S4). Notably, diet supplemented with PM silage increased the concentrations of linoleic acid in milk (LA, C18:2n6, *p* = 0.019), α-linolenic acid (ALA, C18:3n3, *p* = 0.002), arachidonic acid (ARA, C20:4n6, *p* = 0.036), dihomo-γ-linolenic acid (DGLA, C20:3n6, *p* = 0.007), erucic acid (C22:1n9, *p* = 0.001), eicosapentaenoic acid (EPA, C20:5n3, *p* = 0.010), polyunsaturated fatty acid (PUFA) (*p* = 0.049), and omega-6 fatty acid (*p* = 0.001), and decreased the ratio of n6 to n3 (*p* < 0.001) in milk (Table 1). Correspondingly, dairy cows supplemented with PM silage had higher erucic acid (C22:1n9, *p* = 0.011), DGLA (C20:3n6, *p* = 0.004), ARA (C20:4n6, *p* = 0.020), PUFA (*p* = 0.037), and omega-3 fatty acid yield in their milk than dairy cows with a normal diet (Table S5).

### 2.2. Fatty Acid Profile in Blood Plasma

In blood plasma, the concentrations of eicosadienoic acid (EDA, C20:2n6, *p* < 0.001), UFA (*p* = 0.005), docosapentaenoic acid (DPA, C22:5n3, *p* = 0.045), and omega-6 fatty acid (*p* = 0.047) were significantly higher in dairy cows supplemented with PM silage than in those on the normal diet (Table 2). The lower concentration of myristoleic acid (C14:1n5, *p* = 0.036) was observed in the plasma of dairy cows supplemented with PM silage. Notably, an increasing trend was also observed in the concentrations of LA (C18:3n3, *p* = 0.063), ARA (C20:4n6, *p* = 0.065), omega-3 fatty acid (*p* = 0.060), and UFA (*p* = 0.082) in dairy cows supplemented with PM silage.

### 2.3. Fatty Acid Profile and Fermentation Parameters in Rumen Fluid

According to the results of rumen fermentation parameters, a higher concentration of butyrate (*p* = 0.046), isovalerate (*p* = 0.002), and NH_3_-N (*p* < 0.001) and lower pH (*p* < 0.001) were observed in the rumen fluid of dairy cows supplemented with PM silage (Table 3). From the results of a medium- and long-chain fatty acid profile, a diet supplemented with PM silage increased the concentrations of oleic acid (C18:1n9, *p* = 0.038), LA (*p* = 0.030), ALA (*p* < 0.001), eicosatrienoic acid (ETA, C20:3n3, *p* = 0.002), ARA (*p* = 0.045), erucic acid (C22:1n9, *p* = 0.001), UFA (*p* = 0.020), PUFA (*p* = 0.013), and omega-3 (*p* < 0.001) and omega-6 (*p* = 0.028) fatty acids in the rumen fluid of dairy cows supplemented with PM silage. Additionally, diet supplemented with PM silage also increased concentrations of C16:0 (*p* = 0.030), C17:0 (*p* = 0.002), C21:0 (*p* < 0.001), C22:0 (*p* = 0.036), C24:0 (*p* = 0.003), and SFA (*p* = 0.034) and decreased the ratio of n6 to n3 (*p* = 0.039) in rumen fluid of dairy cows (Table 4).

### 2.4. The Dynamics of Ruminal Bacterial Communities

**Figure 1:**
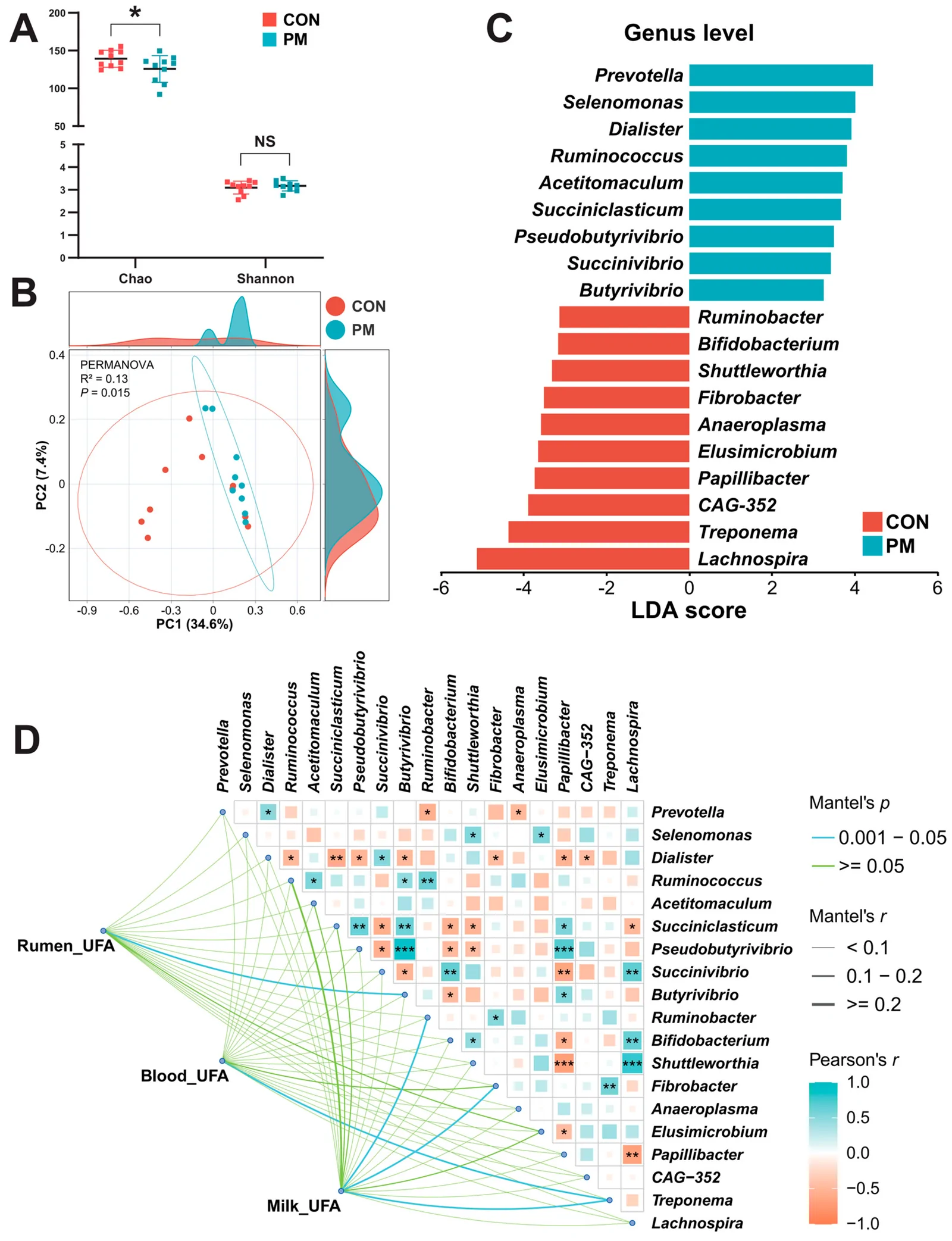
Analysis of rumen bacterial communities based on 16S rRNA sequencing. (**A**) shows the alpha diversity index of rumen bacteria (Chao & Shannon); (**B**) shows the beta diversity analysis of bacterial communities based on permutational multivariate analysis of variance (PERMANOVA); (**C**) shows the linear discriminant analysis at the genus level of rumen bacteria; and (**D**) shows the analysis of Pearson correlation between rumen bacteria and the Mantel-test analysis between rumen bacteria and UFA in rumen, blood, and milk. In the figure, * is *p* < 0.05, ** is *p* < 0.01, and *** is *p* < 0.001. These plots were generated using the CNSknowall platform (https://cnsknowall.com).

The 16S rRNA gene amplicon sequencing was conducted, and a total of 1,352,310 raw reads (67,616 ± 26,819; mean ± standard deviation) and 1,120,390 (56,020 ± 22,724) clean reads were obtained. The clean reads were assigned to 4972 ASVs, which were classified to 17 phyla, 102 families, and 226 genera. The results of bacterial alpha diversity testing showed that the Chao index was significantly lower in the PM group than the CON group (Figure 1A), and beta diversity (Figure 1B) showed a significant difference between the two groups (PERMANOVA, *p* = 0.015). Linear discriminant analysis showed the ranking of important ruminal bacteria (LDA score > 3, *p* < 0.05) in the CON and PM groups at genus level (Figure 1C). The relative abundances of genera *Prevotella*, *Selenomonas*, *Dialister*, *Ruminococcus*, *Acetitomaculum*, *Succiniclasticum*, *Pseudobutyrivibrio*, *Succinivibrio*, and *Butyrivibrio* were higher in dairy cows supplemented with PM silage, while the relative abundances of genera *Ruminobacter*, *Bifidobacterium*, *Shuttleworthia*, *Fibrobacter*, *Anaeroplasma*, *Elusimicrobium*, *Papillibacter*, CAG-352, *Treponema*, and *Lachnospira* were lower in the PM group (Figure 1C). The results of a Mantel-test matrix analysis based on the Bray–Curtis distance indicated that the relative abundances of *Butyrivibrio*, *Ruminobacter*, *Fibrobacter*, and *Treponema* were significantly correlated with the significantly changed UFAs in rumen fluid, plasma, and milk (Figure 1D). The Pearson correlation analysis between ruminal bacterial genera indicated that the relative abundances of *Ruminobacter* and *Treponema* were significantly positively correlated with the relative abundance of *Fibrobacter* (Figure 1D, r > 0.5, *p* < 0.05).

### 2.5. The Alteration of Ruminal Microbial Functions

**Figure 2:**
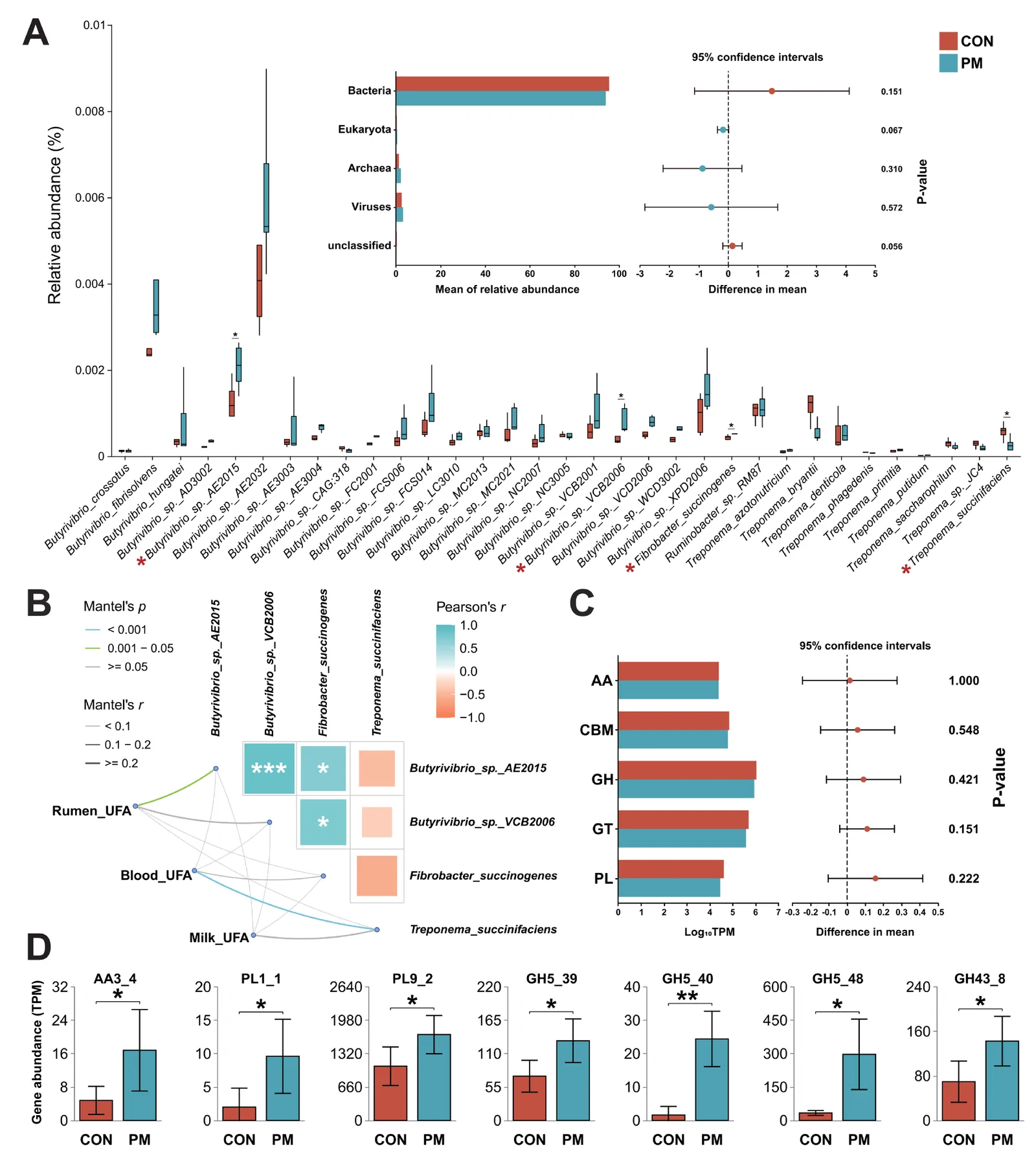
Analysis of rumen microorganisms and carbohydrate-active enzymes based on metagenomics. (**A**) shows the analysis of rumen microbial differences at the domain and species level; (**B**) shows the Pearson correlation between rumen microbial species and the Mantel-test analysis between rumen microbial species and UFA in rumen, blood, and milk; (**C**) shows the two-group comparison of ruminal CAZymes at the class level; and (**D**) shows the two-group comparison of ruminal CAZymes at the subfamily level. * indicates a significant difference (*p* < 0.05, ** *p* < 0.01 and *** *p* < 0.001).

Further metagenomics showed that a total of 993,512,310 raw reads (99,351,231 ± 5,042,102) were obtained; after quality control and removal of the host genes, a total of 986,657,000 optimized reads (98,665,700 ± 5,051,293) were obtained. After reads assembly, a total of 13,630,254 assembled contigs (1,363,025 ± 181,428) were obtained, and these contigs were classified into 134 phyla, 1194 families, 3112 genera, and 15,664 species. No differences were observed in the relative abundance of ruminal bacteria, eukaryota, archaea, and viruses between the CON and PM groups (Figure 2A). In genera *Butyrivibrio*, *Fibrobacter*, and *Treponema*, the relative abundance of species *Butyrivibrio* sp. AE2015, *Butyrivibrio* sp. VCB2006, *Fibrobacter succinogenes*, and *Treponema succinifaciens* was significantly higher in dairy cows supplemented with PM silage (Figure 2A, *p* < 0.05). The results of the Mantel-test matrix analysis based on Bray–Curtis distance showed that ruminal *Butyrivibrio* sp. AE2015 was significantly correlated with UFAs in rumen, and ruminal *Treponema succinifaciens* was significantly correlated with UFAs in plasma (Figure 2B). The Pearson correlation analysis showed that the relative abundance of ruminal *Fibrobacter succinogenes* was significantly positively correlated with the relative abundances of *Butyrivibrio* sp. AE2015 and *Butyrivibrio* sp. VCB2006 (r > 0.7, *p* < 0.05), and the relative abundance of *Butyrivibrio* sp. VCB2006 was significantly positively correlated with the relative abundance of *Butyrivibrio* sp. AE2015 (r = 0.87, *p* < 0.001).

### 2.6. Metabolic Functions of Ruminal Microbiota

**Figure 3:**
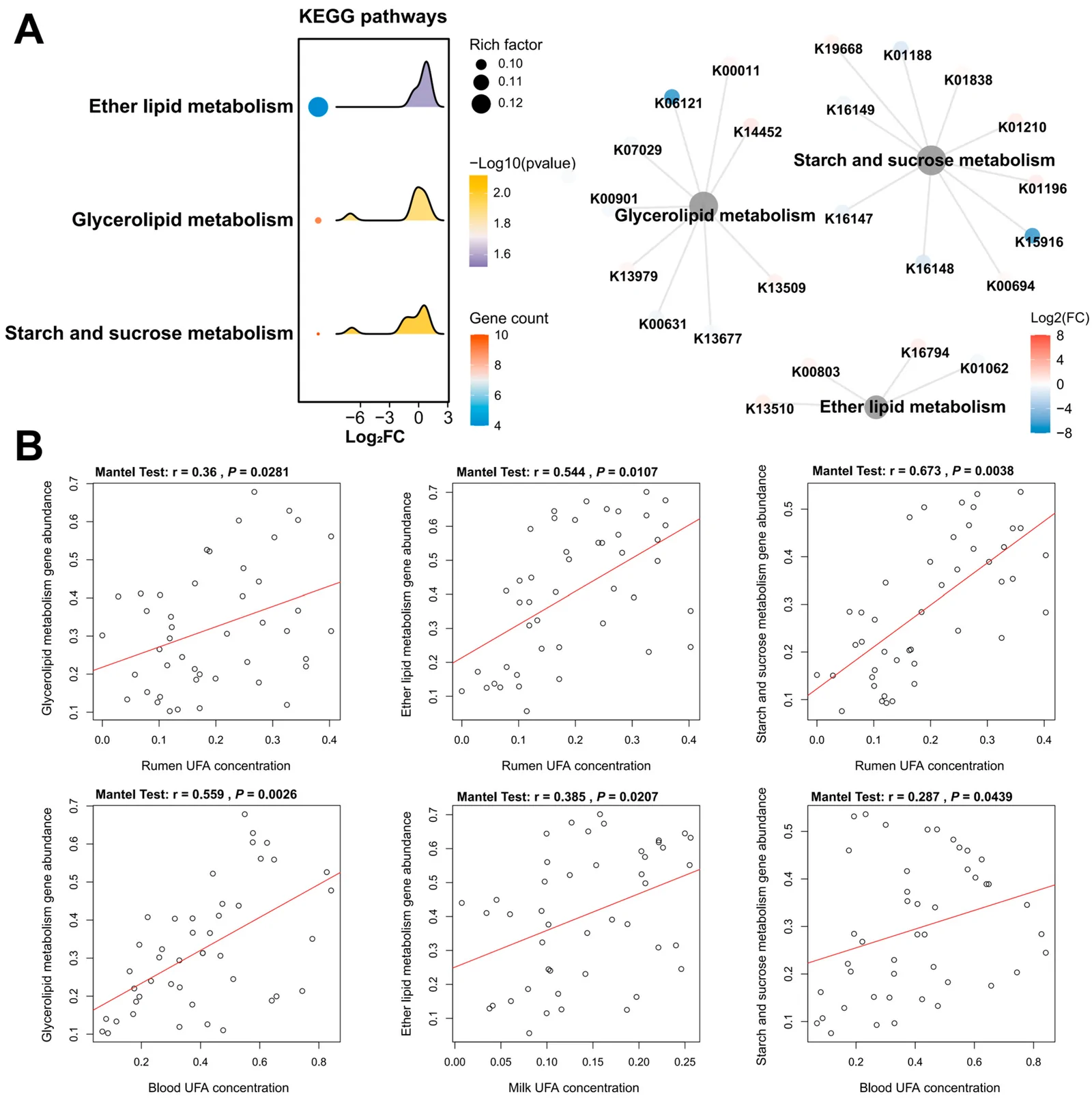
Analysis of KEGG enriched pathways based on metagenomics and the correlation matrix of the Mantel test. (**A**) shows the KEGG pathways related to lipid and carbohydrate metabolism. The mountain-range graph shows the distribution of fold change (FC) of the significantly changed genes in the pathways, while the network graph displays all the significantly changed genes in the pathways. (**B**) shows the analysis of the Mantel-test (Bray–Curtis distance matrix) correlation between UFA (in rumen, blood, and milk) and significantly altered genes in the pathways. The figure depicts the significantly changed UFA and the significantly changed genes in the pathways.

In order to clarify the impact of PM silage on the metabolic functions of rumen microbiota, the microbial functional genes were analyzed. The carbohydrate-active enzymes (CAZymes) profile had no difference between the two groups at class level (Figure 2C). At the subfamily level, diet supplemented with PM silage increased the relative abundance of AA3_4, PL1_1, PL9_2, GH5_39, GH5_40, GH5_48, and GH43_8 (Figure 2D, *p* < 0.05). KEGG functional analysis showed that “ether lipid metabolism”, “glycerolipid metabolism”, and “starch and sucrose metabolism” were significant pathways in dairy cows supplemented with PM silage (Figure 3A). In the ether lipid metabolism pathway, the *LPCAT1_2* (K13510), *AGPS* (K00803), and *PAFAH1B1* (K16794) gene abundances were significantly up-regulated (*p* < 0.05), while *PLA2G7* (K01062) gene abundance was significantly down-regulated in dairy cows supplemented with PM silage (Figure 3A). In the glycerolipid metabolism pathway, the AKR1B (K00011), LIPF (K14452), yahK (K13979), and AGPAT1_2 (K13509) gene abundances were significantly up-regulated (*p* < 0.05), while dhbC (K06121), dgs (K13677), dgkA (K00901), plsB (K00631), and dagK (K07029) gene abundances were significantly down-regulated in dairy cows supplemented with PM silage (Figure 3A, *p* < 0.05). In starch and sucrose metabolism, the CBH2 (K19668), bcsA (K00694), AGL (K01196), E3.2.1.58 (K01210), and pgmB (K01838) gene abundances were significantly up-regulated (*p* < 0.05), while the K16149, glgM (K16148), glgE (K16147), pgi-pmi (K15916), and E3.2.1.21 (K01188) gene abundances were significantly down-regulated in dairy cows supplemented with PM silage (Figure 3A, *p* < 0.05). The Bray–Curtis-distance-based Mantel-test correlation matrix (Figure 3B) shows that the gene abundance in the “ether lipid metabolism pathway” was associated with UFA levels in the rumen and plasma (r = 0.559, *p* = 0.0281), the gene abundance in the glycerolipid metabolism pathway was associated with UFA levels in the rumen and milk (r = 0.544, *p* = 0.0107), and the gene abundance in the starch and sucrose metabolism pathway was associated with UFA levels in the rumen and plasma (r = 0.287, *p* = 0.0439).

### 2.7. Ruminal Metabolites and Omics-Integrative Analysis

**Figure 4:**
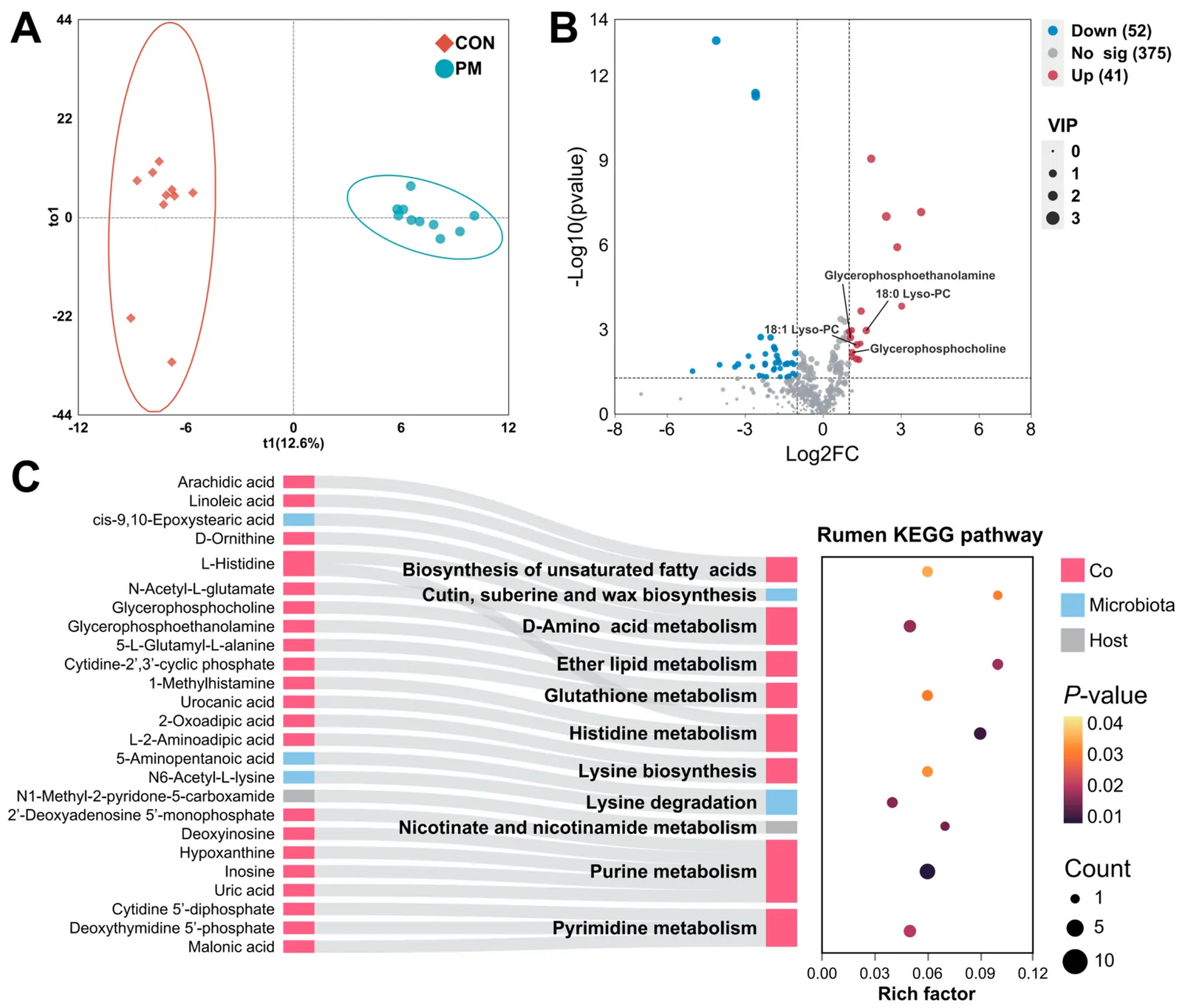
Metabolomics analysis shows the alteration of metabolites in rumen of dairy cows with paper mulberry silage. (**A**) shows the OPLS-DA analysis and permutation test of rumen metabolite in cation and anion. (**B**) shows the volcano plot of the significantly altered metabolites (VIP > 1, |Log_2_FC| > 1). (**C**) shows the Sankey bubble plot of KEGG pathway enrichment analysis. The origins of metabolites and pathways were shown with different colors. ‘Co’ means microbiota-and-host-derived metabolite or pathway, ‘Microbiota’ means microbiota-derived metabolite or pathway, and ‘Host’ means dairy-cow-derived metabolite or pathway.

**Figure 5:**
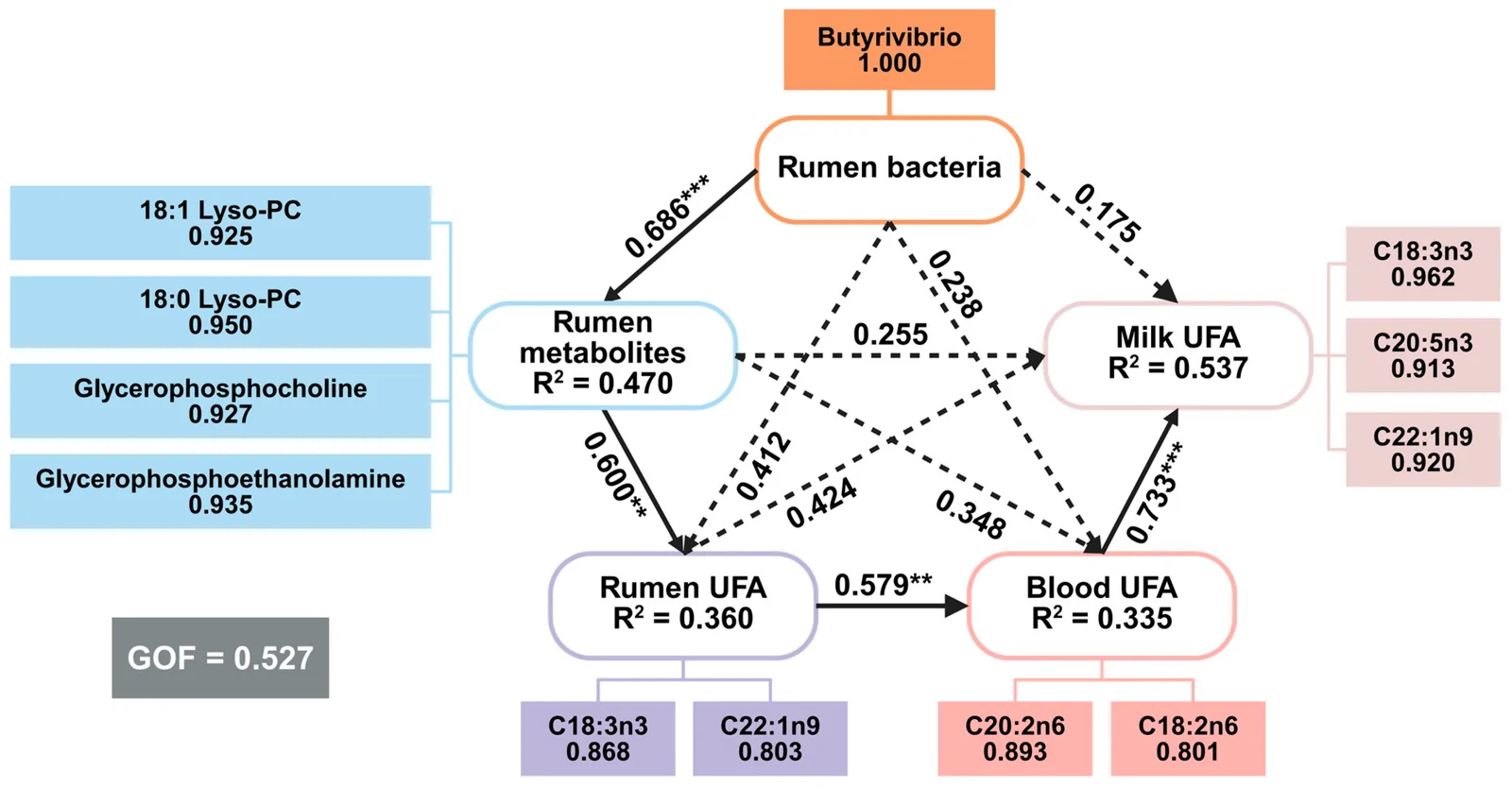
The PLS-PM model describes the hierarchical influence of rumen microbiota, rumen metabolites, rumen UFA, blood UFA, and milk UFA in dairy cows with dietary PM silage. The model was constructed using datasets of significantly changed rumen microorganisms and metabolites and UFA in rumen, blood, and milk. The key factors with a load > 0.8 are presented in the figure. The dotted lines in the figure represent indirect effect, while the solid lines represent direct effect. ** *p* < 0.01 and *** *p* < 0.001.

Rumen non-targeted metabolomics was conducted to clarify how ruminal metabolites affect the metabolism of ruminal UFAs. Orthogonal partial least squares discriminant analysis (OPLS-DA) revealed a clear separation in ruminal metabolomic profiles between the PM and CON groups (Figure 4A). The model exhibited good explanatory and predictive performance (R^2^X = 0.556, R^2^Y = 0.974, and Q^2^ = 0.799), with its robustness further supported by 200 permutation tests (P_R_^2^_Y_ = 0.005) and CV-ANOVA (*p* = 0.002), suggesting a low risk of overfitting (Supplementary Figure S1). A total of 468 metabolites were identified; 41 metabolites were significantly up-regulated and 52 metabolites (VIP > 1, |Log_2_FC| > 1, *p* < 0.05) were significantly down-regulated in dairy cows supplemented with PM silage (Figure 4B). Further KEGG enrichment analysis showed that 25 metabolites were enriched into 11 pathways, including “biosynthesis of unsaturated fatty acid”, “cutin, suberine, and wax biosynthesis”, “D-amino acid metabolism”, “ether lipid metabolism”, “glutathione metabolism”, “histidine metabolism”, “lysine biosynthesis”, “lysine degradation”, “nicotinate and nicotinamide metabolism”, “purine metabolism”, and “pyrimidine metabolism” (Figure 4C). Lastly, a partial least squares path mode (PLS-PM) based on ruminal microbiota, metabolites, UFAs, and plasma and milk UFA datasets was constructed to integrate multi-omics and phenomics (Figure 5). In brief, ruminal *Butyrivibrio* positively affected ruminal UFAs via ruminal 18:1 lyso-PC, 18:0 lyso-PC, glycerophosphocholine, and glycerophosphoethanolamine. Ruminal UFAs positively affected milk UFAs via blood plasma UFAs.

## 3. Discussion

In this study, we explored the effect of diet supplemented with PM silage on milk FA composition and the potential mechanism behind this. In the present study, the PM-silage-based diet did not affect the feed intake and lactation performance of dairy cows, which is consistent with previous studies [18,21]. Therefore, we further studied the potential mechanisms of PM silage enhancing milk PUFA concentration in dairy cows.

It is reported that mulberry fiber can act as a prebiotic, improving the UFA profile in eggs via the gut–liver axis and increasing acetate and propionate concentration in the gut of laying hens [22]. In previous dairy cow studies, diet supplemented with 15% PM silage has significantly increased the PUFA and oleic acid concentration in milk [20]. The higher ruminal acetate, butyrate, and UFA concentrations in the milk of dairy cows in this study are consistent with the results from Hu et al. and Si et al. The fiber from the PM may be able to reshape the fermentation environment of the gastrointestinal tract and the metabolism of UFA in the diet due to its ease of degradation and utilization by microorganisms in the gastrointestinal tract [22]. Additionally, it has been reported that flavonoid from PM may be one of the key factors to regulate host lipid metabolism and increase the PUFA concentration in adipose tissue of finishing pigs via PPARγ–LXRα–ABCA1 signaling [23]. Therefore, we quantified the flavonoid in both the CON diet and the PM-silage-based diet (Table S6), and a higher concentration of total flavonoid was observed in the PM silage than the CON diet. Plant-derived flavonoid has been reported to effectively increase the proportion of oleic acid, CLA, ALA, and MUFA in the milk of dairy cows [24]. Therefore, PM silage may exert multiple effects via the higher flavonoid content and unique dietary fiber properties to regulate the metabolic functions of ruminal microorganisms and reshape the fermentation environment.

Based on the above results and assumptions, we further investigated the changes in rumen microbial communities and functions of dairy cows supplemented with PM silage through 16S rRNA gene sequencing and metagenomics. Firstly, the changed relative abundance of *Selenomonas*, *Butyrivibrio*, and *Ruminococcus* is reported to be involved in rumen UFA metabolism (lipolysis and biohydrogenation) [25,26]. This indicates that supplementing PM silage in the diet affects the content of different UFAs in the rumen by modulating the relative abundance of the core ruminal microorganisms involved in UFA metabolism. Furthermore, the changed relative abundance of ruminal *Prevotella*, *Fibrobacter*, *Selenomonas*, *Butyrivibrio*, *Ruminococcus*, *Lachnospira*, *Pseudobutyrivibrio*, *Acetitomaculum*, *Succinivibrio*, *Bifidobacterium*, *Treponema*, and *Ruminobacter* indicates a reshaped rumen fermentation environment, because they are reported to be widely involved in fiber degradation and short-chain fatty acid metabolism in the rumen [27]. Among these ruminal microorganisms, the *Fibrobacter* associated with the increase of UFAs in milk possesses a unique ability to extensively degrade fibers [27]. This indicates that the PM fibers influence the content of milk UFAs by regulating the fermentation capacity of the rumen. More importantly, *Butyrivibrio*, which is associated with the increase of rumen UFAs, has been widely reported to be the core microorganism involved in the rumen UFA metabolism [25,26,28]. *Butyrivibrio* has a complete functional enzyme system for hydrogenating UFA, including the isomerase and reductase that isomerize and reduce LA [25,29,30]. In addition, *Butyrivibrio* produces large amounts of butyrate when metabolizing diet carbohydrates, which explains the significant up-regulation of ruminal butyrate content [31]. Higher relative abundance of ruminal *Acetitomaculum* also explained a higher ruminal acetate content [27].

Subsequently, we analyzed how PM silage reshapes rumen microbial functions. The up-regulated gene abundance of AA3_4, PL1_1, PL9_2, GH5_39, GH5_40, GH5_48, and GH43_8 indicates that the PM fibers effectively drive the efficient degradation of cellulose, hemicellulose, and pectin in the rumen [32]. It has been reported that the degree of hydrogenation of UFAs can be significantly reduced when they are esterified into phospholipids in the rumen [33], because phospholipids can be integrated into rumen bacteria as components of the cell membrane and form spatial barriers, reducing the accessibility of enzymes, making it difficult for them to be hydrolyzed by rumen lipases and hydrogenated by hydrogenase [33,34,35]. Based on the results of rumen-metagenome-based KEGG pathway enrichment analysis, the “glycerolipid metabolism” and “ether lipid metabolism” are thereby considered the vital pathways regulated by the PM-silage-based diet. In particular, the AGPS (K00803) is the rate-limiting enzyme that catalyzes the formation of alkyl glycerol phosphates during the synthesis of ether lipids, which can promote the synthesis of ether lipids and increase the content of ether phospholipids in the membrane [36]. PAFAH1B1 (K16794) can hydrolyze PAF and oxidized phospholipids, maintaining membrane stability [37]. PLA2G7 (K01062) can regulate the lipid metabolism homeostasis by hydrolyzing short-chain sn-2 ether phospholipids [37]. LPCAT1_2 (K13510) participates in the remodeling of membrane phospholipids through the Lands cycle, catalyzing the reaction between lysophosphatidylcholine (LPC) and acyl-CoA, generating saturated phosphatidylcholine (PC) [38]. The Mantel-test correlation matrix from our results also further confirmed that the functional gene abundance in the “ether lipid metabolism” pathway potentially regulates the UFAs in the rumen and milk. Further results from the rumen metabolome confirmed the result from the metagenome, showing that up-regulated glycerophosphoethanolamine and glycerophosphocholine, which are involved in rumen UFA metabolism, were enriched in the “ether lipid metabolism” pathway.

Beyond its potential involvement in ruminal lipid metabolism, PM silage may serve as an alternative forage resource that contributes dietary protein. Its feeding value depends partly on the plant material used, as PM leaves have higher crude protein concentrations and greater digestibility than stems [39]. In cows with low milk yields, replacing alfalfa hay with PM silage improved antioxidant capacity without significant differences in dry matter intake or milk yield, although apparent protein and fiber digestibility decreased [18]. Another study indicates that replacing rice straw with PM silage in the diet while concurrently reducing the amount of soybean meal can lower feeding costs; this demonstrates the potential economic value of this feed under specific rearing conditions and price environments [40]. Together with the milk fatty acid and ruminal microbial profiles observed here, this evidence suggests potential applications in nutritional management.

In addition to the above-discussed observation, there were still several limitations in this study. Firstly, the present study evaluated endpoint responses to a 56-day sequential feeding regimen comprising 9% PM silage during days 1–28 and 18% during days 29–56. There is a lack of information regarding the individual effects of specific inclusion rates, as well as whether the effects on animals remain stable when the feeding period for PM is extended. The limited sample size and single-endpoint sampling design restrict assessment of PLS-PM model stability and do not establish the temporal sequence or causal mediation underlying the proposed relationships. The estimated paths may also be sensitive to variable selection and model specification. Longitudinal studies with repeated sampling and validation in independent feeding trials are therefore needed to assess the temporal ordering and reproducibility of these associations. Targeted mechanistic experiments, such as controlled microbial perturbation and fatty acid isotope tracing, would further help determine whether the implicated microorganisms and metabolites contribute to ruminal UFA metabolism and FA transfer into milk. The FA metabolism in the gut–liver axis was not explored in this study. Therefore, the significant metabolic role of the gut–liver axis in the post-rumen metabolism of UFAs still requires further investigation to be confirmed. Due to the lack of data on methane, hydrogen, and carbon dioxide emissions, the significant impact of rumen hydrogenation on UFAs has not been thoroughly discussed in this study. The observed positive effects of PM silage on the milk PUFA concentrations may derive from the combined effect of flavonoid and special dietary fiber, which can effectively reshape ruminal microbial function and metabolite profile.

## 4. Materials and Methods

### 4.1. Animal Ethics Statement

This study was approved by the Ethical Committee of the College of Animal Science and Technology of China Agricultural University, Beijing, China (protocol number: 2013-5-LZ). All animals involved in the trial were handled in accordance with applicable institutional and national guidelines for animal use and welfare.

### 4.2. Animal Experimental Design and Sample Collection

Animal experimental design was consistent with our previous study [19]. In brief, 30 healthy Holstein dairy cows with similar body weights (650 ± 37 kg), days in milk (90 ± 15 days), milk yields (36.0 ± 1.2 kg/day), and parity (2.0 ± 0.4) were randomly assigned to either a control (CON) group or a PM group. Both groups were housed in the same barn in opposing pens and managed under identical feeding protocols. Cows in the CON group received no PM silage throughout the experimental period. In contrast, cows in the PM group were fed a diet containing 9% PM silage for the first 28 days, followed by a diet containing 18% PM silage for the subsequent 28 days. Daily feed intake for each group was calculated based on the difference between the total weight of feed provided and the weight of the feed remaining. Dietary composition and nutritional levels are detailed in Supplementary Table S1. During the experiment, the barn utilized a combination of natural and artificial lighting. Artificial lights were switched on only during early morning and evening milking, feeding, and routine inspections; at night, the main lights were turned off, leaving only dim aisle lights to ensure that the cows could rest adequately. The total daily duration of lighting was maintained at 14 to 16 h. Regarding temperature control, the facility was a semi-open, loose-housing barn rather than a fully enclosed, climate-controlled environment. Natural ventilation was regulated via roof vents and adjustable side curtains, while mechanical fans were used for cooling during periods of higher ambient temperatures. The average daily indoor temperature was maintained between 10 °C and 24 °C. All animals were fed twice daily (at 06:00 and 18:00) and milked twice daily (at 05:30 and 17:00), with daily milk yields recorded. Throughout the experiment, the animals had ad libitum access to feed and water, and with an average available space of approximately 12 m^2^ per cow. At the conclusion of the experiment, milk samples were collected and stored at −20 °C for fatty acid (FA) composition analysis. Blood samples were collected from the caudal vein prior to the morning feeding. Blood samples were centrifuged at 3500× *g* for 15 min at 4 °C to separate the plasma, which was then stored at −20 °C for fatty acid profile analysis. [41] Rumen fluid samples were collected via an orogastric tube at the time of blood sampling and stored at −80 °C for the analysis of ruminal volatile fatty acids (VFAs) and the microbiota.

### 4.3. Diet Chemical Component, Fatty Acid, and Flavonoid Profile Measurement

The diets in CON and PM were ground and passed through a 1 mm sieve before measurement of chemical components. The contents of dry matter (DM, method 935.29), crude protein (CP, method 990.03), ash (method 942.05), and ether extract (EE, method 920.39) were measured according to the description of the Association of Official Analytical Chemists (Berbert et al.). The contents of neutral detergent fiber (NDF) and acid detergent fiber (ADF) were analyzed according to the procedures of the Van Soest method [42]. The total FA in the diets was extracted using Agilent DB-WAX capillary column (30 m × 0.25 mm × 0.25 μm) gas chromatography as described by Palmquist et al. [43]. The initial temperature of the FA extraction program was 50 °C for 3 min, then heated up to 220 °C at 10 °C/min, and maintained for 20 min. The carrier gas was helium, and the carrier gas flow rate was 1.0 mL/min. The mass spectrometry analysis procedure was as follows: the injection port temperature was 280 °C, the ion source temperature was 230 °C, and the transmission line temperature was 250 °C, with electron impact ionization source, single ion monitoring mode, electron energy 70 eV. Fatty acid methyl ester was identified by comparing peak retention time and characteristic ions. The standard curves of different fatty acid methyl ester (FAME) were used for fatty acid quantification and calibrated using internal standards.

### 4.4. Blood and Milk Fatty Acid Analysis

The milk fat was extracted as described by a previous study [44]. The FA profile of blood and milk were analyzed by a gas–mass spectrometer (GC–MS, Agilent 7890A/5975C, AgilentTechnologies, Inc., Santa Clara, CA, USA) and determined as FAME. In brief, 150 μL of the sample was mixed with 1 mL of chloroform methanol solution in a 2 mL centrifuge tube. After 30 min of ultrasonic treatment, 2 mL of sulfuric-acid–methanol solution (1%) was mixed with the supernatant, and then the mixture was placed in a water bath (80 °C, 30 min) for methyl esterification. After water bath heating, 1 mL of hexane was added for extraction, and 5 mL of pure water was used for washing. Then, 500 μL of supernatant was mixed with 25 μL of methyl salicylate as the internal standard and added into the injection bottle for detection by GC–MS. The injection volume was 1 μL, and the split ratio was 10:1. The separation and identification procedure of FA profile in blood and milk were the same as for the determination of dietary FA profile.

### 4.5. Rumen VFAs and Medium- and Long-Chain Fatty Acid Measurement

The rumen fluid samples were centrifuged at 800× *g* and 4 °C for 10 min, and the supernatant was mixed with 25% (wt/vol) phosphorous acid at a ratio of 5:1 and stored at −20 °C to determine the concentrations of VFAs by gas chromatography (QP2010 ultra, Shimadzu, Kyoto, Japan) as described by Hu et al. [45]. Rumen medium- and long-chain FAs were analyzed as described by Toral et al. [46]. In brief, 200 mg rumen contents were mixed with 4 mL of hexane and isopropanol (3:2, vol/vol). The organic phases were dried under a continuous flow of nitrogen at 45 °C. FAs were transmethyl-esterified using continuous base–acid-catalyzed transesterification. Then, the fatty acid profile was analyzed using the same method applied for the measurement of blood and milk.

### 4.6. DNA Extraction and 16s rRNA Gene Sequencing

The total DNA was extracted from rumen fluid using the repeat bead-beating plus column method [47]. Ten dairy cows with similar baseline milk yields were selected for sample collection and sequencing. The extracted DNA was first assessed on a 1% agarose gel to check its integrity, and then its purity was evaluated using a NanoDrop 2000 UV–Vis spectrophotometer (Thermo Scientific, Wilmington, NC, USA). The hypervariable regions V3–V4 of the bacterial 16S rRNA genes were amplified with primer pairs 338F (5′-ACTCCTACGGGAGGCAGCAG-3′) and 806R (5′-GGACTACHVGGGTWTCTAAT-3′) via an ABI GeneAmp^®^ 9700 PCR thermocycler (ABI, Foster City, CA, USA). The obtained amplicons were pooled in equimolar and paired-end-sequenced on an Illumina MiSeq PE250 platform (Illumina, San Diego, CA, USA). The raw reads were deposited into the NCBI Sequence Read Archive (SRA) database (accession number: PRJNA1152765). Quality control, denoising, removal of chimeric sequences, and generation of amplicon sequencing variants (ASVs) were performed using the QIIME2 plugin DADA2. Taxonomy was performed with the feature classifier command in QIIME2 using ASVs against the SILVA database (version 138). Alpha diversity (Chao & Shannon) and beta diversity (Bray–Curtis distance) were calculated using the scripts implemented in QIIME2 with a depth of 19,720, which was the lowest ASV number in the dataset.

### 4.7. Rumen Metagenomics Sequencing and Data Processing

The qualified DNA was further used to construct a metagenomic library with an insert size of 350 bp and then sequenced on the Illumina NovaSeq platform. Ten dairy cows were selected for 16S rRNA sequencing, and samples were collected and sequenced. Rumen fluid metagenomic sequences were deposited in the National Center for Biotechnology Information (NCBI) Sequence Read Archive (SRA) database (accession number: PRJNA1152765). The raw sequencing reads were quality-controlled by fastp (v0.20.0) to remove reads with an average quality value lower than 20 and less than 50 bp in length. The host genes were also removed from the filtered reads using BWA (v0.7.17), with the dairy cattle (*Bos taurus*) genome as the reference. The obtained high-quality reads from each sample were individually assembled using Megahit (v1.1.1) with minimum length ≥ 300 bp [48,49]. The open reading frame was predicted using Prodigal (v2.6.3) with the default parameters. The obtained genes were clustered using CD-HIT (v4.8.1), and the longest gene in each cluster was used as a representative sequence to construct a non-redundant gene set [50]. The best-hit taxonomy of non-redundant genes was obtained by aligning them against the NCBI NR database by DIAMOND (v 2.0.13) with an e-value cut-off of 10^−5^. In addition, the obtained unique gene set was aligned against the KEGG database Kanehisa using KofamScan (v1.3.0-2) [51]. Gene quantification (transcripts per million, TPM) was performed using Salmon (v1.10.0) [52].

### 4.8. Rumen Metabolomics Analysis

The rumen fluid samples were dissolved at 4 °C, and subsequently 100 μL of the sample was mixed with 400 μL of the precooled methanol–acetonitrile solution (1:1, *v*/*v*), vortexed for 60 s, and left for 1 h at −20 °C. Then, the samples were centrifuged at 14,000× *g*, 4 °C for 20 min using a cryogenic high-speed centrifuge (5430R, Eppendorf, Hamburg, Germany). The supernatant was taken and frozen and dried. The metabolites were separated on an Agilent 1290 Infinity III UHPLC system (Agilent Technologies, Inc., Santa Clara, CA, USA). The column (ACQUITY UPLC BEH Amide 1.7 µm, 2.1 mm × 100 mm) temperature was 25 °C, and the flow rate was 0.3 mL/min. The mobile phases consisted of A) water + 25 mM ammonium acetate + 25 mM ammonia water, and B) acetonitrile. The samples were placed in an autosampler at 4 °C throughout the analysis. In order to avoid any influence caused by the fluctuation of the instrument detection signal, the samples were consecutively analyzed in random order. The quality-controlled samples were inserted into the sample cohort to monitor and evaluate the stability of the system and the reliability of the experimental data. The samples were then separated by UPLC and analyzed by AB Triple TOF 5600 mass spectrometer (AB SCIEX, Framingham, MA, USA) using electrospray ionization (ESI) positive ion and negative ion modes, respectively. The original data were converted into mzxml format by ProteoWizard, and then peak alignment, retention time correction, and peak area extraction were performed by XCMS program. The metabolite structure was identified by exact mass number matching (<25 ppm) and secondary spectrogram matching, and the self-built database was searched. The origin analysis of significantly altered metabolites and KEGG pathway enrichment were conducted using Metorigin 2.0 [53].

### 4.9. Statistical Analysis

The concentration of VFAs in rumen fluid and the concentrations of FA in rumen fluid, blood, and milk were compared between the two groups using t-test in SAS (v9.4, SAS Institute Inc., Cary, NC, USA) when the data followed normal distribution. The bacterial Chao and Shannon indices between the two groups were compared using Wilcoxon rank sum test, and the *p* values were corrected by false discovery rate. The Bray–Curtis-distance-based microbial β diversity between the two groups was compared using the permutational multivariate analysis of variance method with the R package “vegan” (v4.4.1). The significance of the bacterial difference was tested by linear discriminant analysis (|LDA| > 3). The Mantel tests between ruminal microbiota, UFAs, and functional genes were analyzed using the linkET in R package (v4.4.1), and the correlation method between ruminal microbiota was Pearson. For rumen metabolomics analysis, peak areas were scaled to unit variance before orthogonal partial least squares discriminant analysis (OPLS-DA). The OPLS-DA model was validated using 7-fold cross-validation, 200 permutation tests, and CV-ANOVA to assess predictive performance and minimize overfitting [54]. Differential metabolites were screened based on VIP > 1 and *p* < 0.05, with *p* values adjusted using the Benjamini–Hochberg false discovery rate (FDR) procedure across all 468 metabolites [55]. Metabolites with adjusted *p* values (q values) < 0.05 were considered statistically significant after multiple-testing correction. The PLS-PM model was constructed using significantly altered FAs, ruminal genera, and metabolite and functional gene datasets with R package “plspm”. For all analyses, *p* < 0.05 was considered as significant difference. Visualization of the data was completed using the ggplot2 in R package (v4.4.1) and the CNSknowall platform (https://www.cnsknowall.com).

## 5. Conclusions

Integrated rumen 16S rRNA gene sequencing, metagenomics, and metabolomics revealed that diet supplemented with PM silage can effectively reshape rumen microbiota-mediated fermentation and functions and metabolic profile in dairy cows. Specifically, dietary PM silage changed the relative abundance of ruminal core microorganisms such as *Fibrobacter*, *Butyrivibrio*, *Treponema*, and *Ruminobacter*, which were highly correlated with UFA levels in the rumen, blood plasma, and milk of dairy cows. Through an integrated association analysis combining rumen metagenomics and metabolomics, this study identified a strong correlation between “ether lipid metabolism” and the metabolic pathways of unsaturated fatty acids in the rumen. Specifically, functional genes including *AGPS (K00803)*, *PAFAH1B1 (K16794)*, *PLA2G7 (K01062)*, and *LPCAT1_2 (K13510)*, along with metabolites such as glycerophosphoethanolamine and glycerophosphocholine, have been identified as candidate factors involved in regulating the content of unsaturated fatty acids in the rumen and in milk. Our study addressed the positive effects of dietary supplementation with PM silage on milk PUFA concentration and the underlying mechanisms, providing a theoretical foundation for PM silage usage for PUFA-enriched milk production in the dairy industry.

## Figures and Tables

**Table 1:** The effects of dietary paper mulberry silage on milk fatty acid proportion (mg/mL).

Items	Treatment		SEM	*p*-Value
	CON	PM		
C6:0	0.522	0.476	0.014	0.125
C8:0	0.381	0.368	0.009	0.971
C10:0	1.027	1.055	0.022	0.247
C12:0	1.256	1.304	0.033	0.247
C13:0	0.060	0.050	0.002	0.070
C14:0	3.454	3.602	0.096	0.247
C15:0	0.442	0.378	0.014	0.002
C16:0	9.364	9.087	0.325	0.631
C17:0	0.178	0.173	0.004	0.705
C18:0	2.555	2.653	0.079	0.559
C20:0	0.036	0.039	0.001	0.302
C21:0	0.015	0.018	0.001	0.009
C22:0	0.018	0.022	0.001	0.004
C23:0	0.011	0.013	0.000	0.031
C24:0	0.012	0.014	0.000	0.037
C14:1n5	0.284	0.255	0.010	0.481
C16:1n7	0.468	0.339	0.022	0.001
C18:1n9	4.864	4.764	0.130	0.393
C18:2n6	0.699	0.789	0.027	0.019
C18:3n3	0.085	0.110	0.004	0.002
C20:1n9	0.014	0.019	0.001	0.073
C20:3n6	0.040	0.051	0.002	0.007
C20:4n6	0.053	0.055	0.002	0.036
C20:5n3	0.007	0.008	0.000	0.010
C22:1n9	0.024	0.027	0.001	0.001
SFA	19.413	19.312	0.558	0.481
UFA	6.440	6.376	0.168	0.353
PUFA	0.884	1.020	0.037	0.049
MUFA	5.574	5.340	0.144	0.796
n3	0.797	0.903	0.030	0.088
n6	0.089	0.118	0.005	0.001
n6/n3	9.061	7.717	0.212	<0.001

SFA: saturated fatty acid. UFA: unsaturated fatty acid. PUFA: polyunsaturated fatty acid. MUFA: monounsaturated fatty acid. CON: diet without paper mulberry silage supplementation, PM: diet supplement with paper mulberry silage. SEM: mean of standard error.

**Table 2:** The effects of dietary paper mulberry silage on plasma fatty acid composition (µg/mL).

Items	Treatment		SEM	*p*-Value
	CON	PM		
C12:0	0.257	0.260	0.006	0.798
C13:0	0.168	0.168	0.002	1.000
C14:0	1.673	1.839	0.142	0.572
C15:0	2.406	2.750	0.215	0.440
C16:0	61.254	64.657	5.273	0.756
C17:0	4.428	4.988	0.405	0.505
C18:0	2.619	4.441	8.744	0.949
C20:0	0.394	0.394	0.030	0.571
C21:0	0.686	0.955	0.008	0.878
C22:0	0.484	0.502	0.011	0.261
C23:0	0.907	2.142	0.007	0.091
C24:0	98.646	135.581	0.010	0.137
C14:1n5	0.411	0.327	0.020	0.036
C15:1n5	0.360	0.361	0.010	0.961
C16:1n7	2.230	1.941	0.189	0.459
C17:1n7	0.941	1.000	0.063	0.654
C18:1n9	18.962	22.911	2.077	0.356
C18:1tn9	5.350	5.905	0.480	0.577
C18:2n6	17.830	33.264	4.061	0.063
C18:2ttn6	0.456	0.450	0.033	0.930
C18:3n6	4.227	4.647	0.350	0.564
C20:1n9	0.482	0.517	0.009	0.465
C20:2n6	0.114	0.119	0.098	<0.001
C20:3n8	0.274	0.937	2.371	0.305
C20:4n6	2.101	2.131	0.491	0.065
C20:5n3	21.163	26.202	0.186	0.938
C22:1n9	0.425	0.451	0.154	0.449
C22:2n6	1.939	2.180	0.072	0.931
C22:4n6	4.938	9.597	1.374	0.280
C22:5n3	0.850	0.863	1.155	0.045
C22:5n6	4.431	5.465	0.145	0.623
C22:6n3	7.857	9.745	0.466	0.279
C24:1n9	0.745	0.776	0.413	0.256
MUFA	31.22	36.89	3.102	0.376
PUFA	31.217	36.887	6.245	0.116
SFA	68.561	88.341	14.759	0.852
UFA	183.676	189.417	10.577	0.082
n3	9.370	15.061	1.513	0.060
n6	34.797	55.301	5.168	0.047
n6/n3	3.932	3.945	0.270	0.982

SFA: saturated fatty acid. UFA: unsaturated fatty acid. PUFA: polyunsaturated fatty acid. MUFA: monounsaturated fatty acid. CON: diet without paper mulberry silage supplementation, PM: diet supplement with paper mulberry silage. SEM: mean of standard error.

**Table 3:** The effects of dietary paper mulberry silage on the rumen volatile fatty acid profile.

Items	CON	PM	SEM	*p*-Value
Acetate (mM)	55.19	61.41	1.814	0.073
Propionate (mM)	18.97	21.19	1.446	0.423
Isobutyrate (mM)	0.40	0.54	0.305	0.070
Butyrate (mM)	9.18	11.03	0.648	0.046
Isovalerate (mM)	1.29	1.74	0.077	0.002
Valerate (mM)	1.09	1.20	0.110	0.375
TVFA (mM)	86.12	96.90	3.242	0.075
pH	6.61	5.98	0.080	<0.001
Acetate:Propionate	3.05	3.07	0.154	0.971
NH_3_-N (mg/dL)	16.65	21.37	0.752	<0.001

TVFA: total volatile fatty acid. NH_3_-N: ammonia nitrogen.

**Table 4:** The effects of dietary paper mulberry silage on rumen fatty acid profile (μg/mL).

Items	Treatment		SEM	*p*-Value
	CON	PM		
C10:0	3.477	1.878	0.659	0.496
C12:0	7.419	8.264	0.401	0.472
C13:0	2.147	3.176	0.487	0.344
C14:0	23.630	23.428	1.816	0.970
C15:0	16.246	21.646	2.161	0.222
C16:0	326.175	440.734	26.901	0.030
C17:0	6.305	9.553	0.575	0.002
C18:0	730.432	894.470	45.440	0.071
C20:0	10.193	12.826	0.884	0.143
C21:0	4.876	8.105	0.460	<0.001
C22:0	6.662	9.436	0.670	0.036
C23:0	3.031	3.422	0.191	0.324
C24:0	7.762	10.747	0.544	0.003
C16:1n7	2.349	2.327	0.127	0.472
C18:1n9	96.386	122.526	7.043	0.038
C18:2n6	84.005	111.455	6.377	0.030
C18:3n3	11.066	17.525	1.028	<0.001
C20:1n9	3.120	3.685	0.289	0.791
C20:4n6	2.652	3.165	0.228	0.045
C20:3n3	1.380	2.638	0.211	0.002
C22:1n9	5.739	7.172	0.268	0.001
SFA	1148.357	1447.688	72.048	0.034
UFA	206.698	270.493	13.873	0.020
PUFA	99.102	134.782	7.322	0.013
MUFA	107.594	135.710	7.449	0.031
n3	12.445	20.164	1.148	<0.001
n6	86.656	114.619	6.436	0.028
n6/n3	7.284	5.620	0.398	0.039

SFA: saturated fatty acid. UFA: unsaturated fatty acid. PUFA: polyunsaturated fatty acid. MUFA: monounsaturated fatty acid. CON: diet without paper mulberry silage supplementation, PM: diet supplement with paper mulberry silage. SEM: mean of standard error.

## Data Availability

The data for rumen 16S rRNA gene sequencing and metagenomics are available on the NCBI Sequence Read Archive (SRA) database (PRJNA1152765).

## Supplementary Materials

The following supporting information can be downloaded at: https://www.mdpi.com/article/10.3390/ijms27219940/s1.
