# Supplementary material for: Paper-Mulberry-Silage-Enhanced Lipid Metabolism and Milk Polyunsaturated Fatty Acid Concentration in Dairy Cows: A Multi-Omics Study
Source: Int J Mol Sci. 2026 Nov 6;27(21):9940. doi: 10.3390/ijms27219940 (PMC13764039; doi:10.3390/ijms27219940)
Supplement: Supplementary file 1 [file ijms-27-09940-s001.zip › ijms-4620119-supplementary.pdf]

Paper Mulberry Silage Enhanced Lipid Metabolism and Milk Polyunsaturated Fatty Acids Concentration in Dairy Cows: A Multi-Omics Study

Shuaikang Yang[1†], Weixuan Tang[2†], Yajing Wang[2], Hongjian Yang[2], Wei Wang[3], Zhijun Cao[2], Wei Wang[2], Shengli Li[2], Yueyu Bai[1*], Yangyi Hao[2*]

[1] Institute of Livestock Animal Breeding and Reproduction, School of Life Sciences, Henan University, Zhengzhou, Henan 450046, China.

[2] State Key Laboratory of Animal Nutrition and Feeding, Beijing Engineering Technology Research Center of Raw Milk Quality and Safety Control, College of Animal Science and Technology, China Agricultural University, Beijing 100193, China

[3]School of Applied Science, Taiyuan University of Science and Technology, Taiyuan, 030024, China.

[†] These authors equally contributed to this work.

[*] Corresponding author: Yangyi Hao, E-mail address: haoyangyi0928@163.com, Yueyu Bai, E-mail address: baiyy666@henu.edu.cn

Table S1 Composition and nutrient levels of experimental diets.

Items[1]	Treatment			
	Period I		Period II	
	CON	PM	CON	PM
Ingredients,% DM				
Oat	0.00	0.00	4.10	6.10
Alfalfa hay	6.00	4.00	6.20	0.00
Alfalfa silage	8.60	2.80	7.40	0.00
Paper mulberry silage	0.00	9.00	0.00	18.00
Whole corn plant silage	38.50	38.00	34.70	27.30
Steam-flaked corn	13.60	13.60	12.00	12.60
Whole cottonseed	2.10	2.10	2.10	2.10
Soybean meal	13.20	13.20	15.30	15.30
Corn	9.30	9.30	9.40	9.40
Bran	1.50	1.50	1.50	1.50
Extruded soybean meal	1.10	1.10	1.10	1.10
DDGS	2.10	2.10	2.20	2.20
Sodium bicarbonate	1.10	1.10	1.10	1.10
Magnesium oxide	0.30	0.30	0.30	0.30
Yeast	0.30	0.30	0.30	0.30
Montmorillonite	0.10	0.10	0.10	0.10
Mineral vitamin premix[2]	2.10	2.10	2.10	2.10
Chemical levels,% DM				
DM	50.66	50.28	51.02	50.48
CP	17.10	17.20	17.20	17.20
EE	4.50	4.70	4.60	4.60
Ash	5.90	6.10	5.80	5.90
NDF	29.80	30.10	30.40	30.30
ADF	20.10	20.30	20.40	20.60
NE_L[3] (Mcal/kg)	1.70	1.70	1.70	1.70

[1] DM: dry matter; DDGS: dry distillers grains soluble; CP: crude protein; EE: ether extract; NDF: neutral detergent fiber; ADF: acid detergent fiber; NE_L: net energy for lactation.

[2] Premix provided the following per kg: 6000 mg Mn; 4800 mg Fe;
9000 mg Zn; 2600 mg Cu; 120 mg I2; 80 mg Se; 70 mg Co; 130,000 IU Vitamin A; 336,000 IU Vitamin D; and 465 IU
Vitamin E.

[3] NE_L was calculated using NRC (2001) model.

Table S2 Feed intake and lactation performance of dairy cows supplemented with PM silage.

Item	Treatment		SEM[4]	P-value
	CON	PM		
DMI[1], kg/day	22.9	22.8	0.500	0.680
Milk yield, kg/day	33.9	33.8	0.775	0.764
Milk composition, %				
Fat	3.99	4.15	0.098	0.427
Protein	3.59	3.58	0.073	0.947
Lactose	4.79	5.01	0.102	0.289
Total solid	12.7	13.0	0.246	0.466
MUN[2] (mg/dL)	15.9	17.1	0.341	0.090
SCC[3] (×10[3])	26.1	22.6	5.10	0.736

[1]Dry matter intake.

[2]Milk uric nitrogen.

[3]Somatic cell count.

[4]Standard error of mean.

Table S3 The fatty acid concentration of experimental diets. (mg / g)

Items	Treatment	
	CON	PM
C10:0	0.02	0.02
C12:0	0.06	0.04
C14:0	0.17	0.15
C16:0	11.29	11.34
C17:0	0.11	0.10
C18:0	1.71	1.64
C20:0	0.35	0.32
C21:0	0.01	0.13
C22:0	0.32	0.31
C23:0	0.09	0.07
C24:0	0.31	0.33
C16:1n7	0.15	0.14
C18:1n9	11.05	10.24
C18:2n6	28.42	25.91
C18:3n3	3.23	3.63
C20:1n9	0.14	0.13
C20:2n6	0.02	0.03
C20:3n6	0.01	0.02
C20:3n3	0.00	0.02
SFA	14.44	14.45
UFA	43.03	40.11
PUFA	31.69	29.60
MUFA	11.34	10.51
n6	28.46	25.96
n3	3.23	3.65
n6/n3	8.80	7.12

SFA: saturated fatty acid; UFA: unsaturated fatty acid; PUFA: polyunsaturated fatty acid; MUFA: monounsaturated fatty acid; n6: ω-6 fatty acid; n3: ω-3 fatty acid.

Table S4 Dietary fatty acid intake of dairy cows. (g / d)

Items	Treatment	
	CON	PM
C10:0	0.52	0.36
C12:0	1.42	0.93
C14:0	3.91	3.39
C16:0	258.74	259.92
C17:0	2.62	2.33
C18:0	39.20	37.51
C20:0	8.12	7.36
C21:0	0.20	3.01
C22:0	7.35	7.12
C23:0	1.96	1.58
C24:0	7.13	7.51
C16:1n7	3.38	3.10
C18:1n9	253.37	234.72
C18:2n6	651.58	593.59
C18:3n3	74.13	83.13
C20:1n9	3.15	2.96
C20:2n6	0.48	0.69
C20:3n6	0.33	0.40
C20:3n3	0.00	0.41
SFA	331.16	331.02
UFA	986.42	919.00
PUFA	726.52	678.23
MUFA	259.90	240.78
n6	652.39	594.68
n3	74.13	83.54
n6/n3	8.80	7.12

SFA: saturated fatty acid; UFA: unsaturated fatty acid; PUFA: polyunsaturated fatty acid; MUFA: monounsaturated fatty acid; n6: ω-6 fatty acid; n3: ω-3 fatty acid. The fatty acid intake was calculated by multiplying the concentration of fatty acids in the diet with the average daily feed intake of the cows. CON: diet without paper mulberry silage supplementation, PM: diet supplement with paper mulberry silage.

Table S5 The milk fatty acid yield of dairy cows (g / d)

Items	Treatment		SEM	*P*-value
	CON	PM		
C6:0	17.56	16.49	0.684	0.448
C8:0	12.82	12.77	0.482	0.963
C10:0	34.50	36.73	1.409	0.445
C12:0	42.20	45.52	1.915	0.400
C13:0	2.01	1.73	0.101	0.164
C14:0	9.51	8.82	0.358	0.348
C15:0	14.87	13.18	0.631	0.187
C16:0	15.77	11.91	0.871	0.022
C17:0	5.98	6.02	0.223	0.935
C18:0	2.83	3.84	0.199	0.007
C20:0	0.48	0.65	0.046	0.067
C21:0	1.76	1.92	0.071	0.288
C22:0	0.80	0.95	0.036	0.044
C23:0	0.38	0.46	0.021	0.076
C24:0	115.91	125.81	5.406	0.374
C14:1n5	314.44	317.04	14.745	0.932
C16:1n7	85.56	92.17	3.615	0.375
C18:1n9	162.96	165.60	6.299	0.841
C18:2n6	23.28	27.45	1.141	0.065
C18:3n3	1.21	1.35	0.058	0.238
C20:1n9	0.49	0.62	0.027	0.011
C20:3n6	1.34	1.74	0.075	0.004
C20:4n6	0.59	0.76	0.036	0.020
C20:5n3	0.24	0.28	0.012	0.079
C22:1n9	0.40	0.48	0.024	0.081
SFA	651.38	672.77	27.792	0.711
UFA	215.52	221.72	8.156	0.715
PUFA	29.44	35.52	1.486	0.037
MUFA	186.81	185.55	6.888	0.930
n6	26.54	31.41	1.287	0.056
n3	2.97	4.12	0.213	0.003

SFA: saturated fatty acid; UFA: unsaturated fatty acid; PUFA: polyunsaturated fatty acid; MUFA: monounsaturated fatty acid; n6: ω-6 fatty acid; n3: ω-3 fatty acid. CON: diet without paper mulberry silage supplementation, PM: diet supplement with paper mulberry silage. SEM: Mean of standard error.

Table S6 The chemical composition of flavonoids / isoflavonoids in experimental diets. (ng / mg)

Items	Treatment	
	CON	PM
Catechin	8.70	3.31
Genistein	400.74	405.71
Kaempferol	24.56	11.00
Luteolin	45.70	571.65
Rutin	12.17	7.48
Daidzein	154.13	152.64
Naringin	0.00	0.00
Chrysin	0.09	0.32
Isoquercitrin	7.65	4.24
Cynaroside	1.33	1.41
Apigenin	354.08	1393.89
Biochanin A	1.74	0.14
Daidzin	334.86	218.24
Taxifolin	0.73	0.62
Epicatechin	0.70	0.99
Formononetin	42.53	2.41
Genistin	295.00	230.22
Glycitein	85.26	72.36
Glycitin	38.01	10.86
Liquiritigenin	3.77	0.81
Quercitrin	0.98	0.12
Vitexin	5.87	10.03
Naringenin	11.55	31.01
Kaempferide	0.00	0.00
Myricetin	0.09	0.04
Puerarin	0.11	0.09
Baicalin	0.00	0.00
Diosmin	1.74	0.00
Icariin	0.00	0.00
Fisetin	0.00	0.00
Isovitexin	13.02	20.80
Astragalin	52.35	13.86
Dihydromyricetin	0.00	0.00
Isorhamnetin	14.38	8.87
Sakuranetin	0.00	0.00
Dihydrokaempferol	2.70	1.50
Epigallocatechin	0.06	0.07
Eriodictyol	0.81	5.83
Gallocatechin	0.00	0.00
Apiin	0.20	0.26
Rhoifolin	0.29	0.13
Phloretin	0.03	0.82
Phlorizin	0.08	0.05
Prunin	4.56	4.67
Hesperetin	0.00	0.03
Isoliquiritigenin	9.91	1.35
Quercetin	5.31	2.93
Total	2043.52	3299.20

Table S7. OPLS-DA scores of individual animals (CON, n = 10; PM, n = 10).

Animal ID	Group	Predictive score (t1)	First orthogonal score (to1)
CON1	CON	-7.247	2.841
CON2	CON	-6.634	4.755
CON3	CON	-7.854	9.978
CON4	CON	-7.492	12.426
CON5	CON	-7.097	4.922
CON6	CON	-5.607	5.451
CON7	CON	-8.720	8.149
CON8	CON	-6.785	-32.234
CON9	CON	-6.778	6.148
CON10	CON	-9.067	-22.437
PM1	PM	10.090	0.342
PM2	PM	6.540	6.942
PM3	PM	8.189	-4.799
PM4	PM	5.778	1.801
PM5	PM	7.814	-1.642
PM6	PM	9.284	-3.170
PM7	PM	6.572	-0.627
PM8	PM	7.038	-0.949
PM9	PM	6.126	1.736
PM10	PM	5.851	0.367

Table S8. Validation of the OPLS-DA models (CON vs PM).

Parameter	Model used in the figure	Sensitivity model (log2-transformed)
Transformation	None (peak areas)	log2
Scaling	Unit variance	Unit variance
Predictive/orthogonal components	1/2	1/3
R2X of predictive component (t1)	0.126	0.13
R2X(cum)	0.556	0.615
R2Y(cum)	0.974	0.992
Q2(cum)	0.799	0.847
RMSEE	0.091	0.053
Cross-validation	7-fold	7-fold
Number of response permutations	200	200
Permuted models with R2Y >= observed	0	3
Permuted models with Q2 >= observed	0	0
Permutation P for R2Y	0.005	0.02
Permutation P for Q2	0.005	0.005
Intercept of permutation regression, R2Y	0.883	0.969
Intercept of permutation regression, Q2	-0.378	-0.314
CV-ANOVA F (df)	6.54 (6, 13)	6.44 (8, 11)
CV-ANOVA P	0.002	0.003
Component-wise statistics of the model in Table S7		
p1: R2X / R2Y / Q2	0.126 / 0.514 / 0.350	
o1: R2X / R2Y / Q2	0.340 / 0.380 / 0.341	
o2: R2X / R2Y / Q2	0.090 / 0.080 / 0.108	

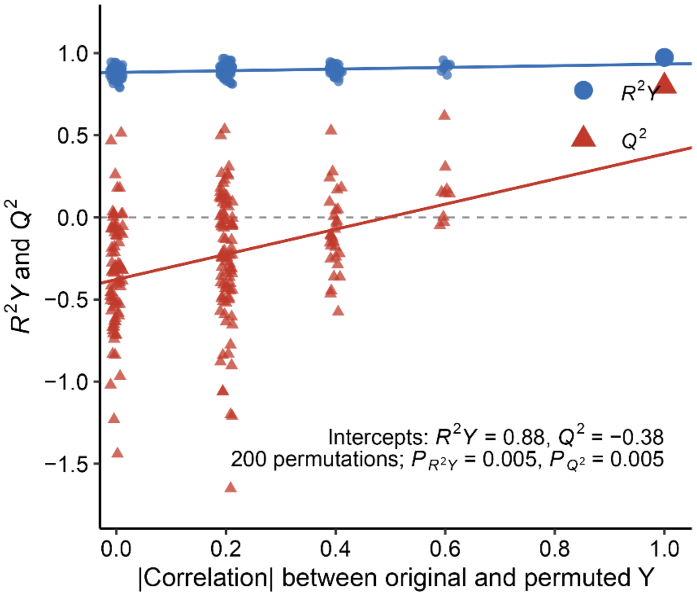

Figure S1 Permutation test plot for OPLS-DA model
